# The coloring mechanism of a novel golden variety in *Populus deltoides* based on the RGB color mode

**DOI:** 10.48130/FR-2021-0005

**Published:** 2021-02-22

**Authors:** Yuru Tian, Shupei Rao, Qianqian Li, Meng Xu, Aike Wang, Hechen Zhang, Jinhuan Chen

**Affiliations:** 1 College of Biological Sciences and Technology, Beijing Advanced Innovation Center for Tree Breeding by Molecular Design, National Engineering Laboratory for Tree Breeding, Beijing Forestry University, 100083 Beijing, China; 2 Yucheng Institute of Agricultural Sciences, Shangqiu, Henan 476000, China; 3 Shangqiu Zhongxing Seedling Planting Co., Ltd, Shangqiu, Henan 476000, China; 4 Horticultural Research Institute, Henan Academy of Agricultural Sciences, Zhengzhou, Henan 450002, China

**Keywords:** poplar, leaf color, chlorophyll, anthocyanin, carotenoids

## Abstract

Compared to flower color and red leaf mutants, the mechanism of yellow leaf formation in woody plants is more complicated and less understood. Poplars are common and widely distributed perennial trees. Here, we obtained a golden leaf mutant poplar variety (JHY) and compared it with its original green leaf poplar (*Populus sp*. Linn. ‘2025’) in terms of phenotype, pigment content, the transcriptomes and metabolomes. Through transcriptome sequencing, we found that the chlorophyll degradation genes, and the genes in anthocyanin metabolism in JHY were up-regulated compared to L2025 and the carotenoid synthesis genes were down-regulated. Further based on HPLC-ESI-MS/MS technology, 16 differentially expressed anthocyanins were identified, 14 reddish anthocyanins of which were significantly up-regulated. According to these results, we proposed a coloring mechanism of JHY based on the RGB color mode. The yellow color of carotenoids and the red color of anthocyanins overlay each other, combined with a reduction in chlorophyll, turning the leaves golden. This study systematic analyzes the causes of golden leaf formation through the omics integration approach, which will provide reference for the breeding of golden leaf trees.

## INTRODUCTION

Leaf color is an essential trait of plants, and ornamental plant cultivation and foliage color will be more diversified (multi-species, multi-color) in the future. Novel plant varieties with colorful leaves will increase in popularity and have a more important role in landscaping. The most attractive ornamental feature of some plant species is their golden leaves which usually occur in autumn^[1]^. Trees with colored leaves not only enrich roadside landscapes, but also provide protection against wind, sand and soil erosion. The color changes in colorful plants are complex, and color traits primarily determined by leaf pigments^[1]^ and correlated metabolic composition^[2]^. Leaf pigments are mainly divided into chlorophyll, carotenoids and anthocyanins. As leaf senescence occurs in autumn, leaf color usually changes from green to yellow or red. This makes color change a recognizable event in the plant life cycle^[3]^. Red colored leaves associated with high levels of anthocyanins have been extensively studied^[4]^. Red leaves have been studied in woody plants such as *Acer rubrum*^[5]^, *Populus deltoides*^[6]^, and *Ginkgo biloba*^[7]^. Compared to red leaf mutants, the mechanism of yellow leaf formation in woody plants is more complicated and less understood.

Chlorophyll is critical for photosynthesis, and alteration in chlorophyll synthesis can result in leaf color variations^[1, 8]^. The chlorophyll metabolic pathway in higher plants consists of three steps: chlorophyll biosynthesis, chlorophyll cycle (interconversion of chlorophyll a and chlorophyll b), and chlorophyll degradation^[9−11]^. Chlorophyll biosynthesis involves a series of enzymatic reactions, and the absence of any reaction will reduce the content of chlorophyll, and result in green-deficient leaf^[12]^. Carotenoid is the generic term for carotenes and xanthophylls, which are terpenoids. Carotenoids are critical for photosynthesis and contribute to the yellow and orange hues of most plants^[13]^. *Cis*-lycopene, carotene and xanthophylls are the main carotenoid pigments in plant photosystems^[14]^. An EMS-induced yellow young leaf mutant *C777* of *Cucumis sativus*, results in a decrease in the expression of chlorophyll-related genes (*CsHD*) and yellow leaf color^[15]^. *Lrysl1*, a lethal mutant in yellow seedlings, was found in the self-bred progenies of *Lilium regale*. Chlorophyll and carotenoid contents in the leaves of this mutant were lower than that of those in wild type^[16]^. The effects of chlorophyll synthesis and degradation of plant color have been reported in *Pisum sativum*^[17]^, *Helianthus annuus*^[18]^, and *Oryza sativa*^[19]^. The yellow plant mutant appears to be useful for studying the mechanisms of chlorophyll and carotenoid synthesis.

Anthocyanins, are flavonoids synthesized through the phenylpropane pathway and then transported to the vacuoles and other parts of plants^[20, 21]^. Anthocyanins are divided into six common anthocyanidins, namely cyanidin (magenta), delphinidin (purple-blue, with one more hydroxyl than cyanidin), pelargonidin (orange-red, with one less hydroxyl group than cyanidin), peonidin, petunidin, and malvidin, which are differentiated according to the hydroxyl pattern or methoxy substitutions of the aromatic B ring^[22, 23]^. In *Poplar spp.*, metabolites such as cyanidin, cyanidin 3-*O*-glucoside, and delphinidin 3-*O*-glucoside have been identified^[24]^. In *Ziziphus jujuba*, the anthocyanin content of red-skinned pulp and the genes regulating the enzymes that regulate anthocyanin were significantly up-regulated^[25]^. In *Malus hupehensis*, the total anthocyanin content is consistent with the color change of begonia flowers^[26]^. Anthocyanins are clearly important substances involved in the determination of leaf color.

Poplar is often used as a model system for characterizing tree-specific processes such as dormancy, secondary wood formation, and responses to biotic and abiotic stresses. *P. deltoides* is a biologically and economically important species. It is often used to develop new poplar varieties, because of its rapid growth, good morphology, and easy vegetative reproduction. A number of natural and artificially cultivated varieties of *P. deltoides*, with desirable horticultural traits, such as leaf color variation, are commercially available. Forest scientists have developed many new poplar varieties, such as *Populus sp.* Linn. ‘2025’ originating from *Populus deltoides*^[27]^. A poplar variety with golden leaves (JHY) is a bud mutation from *Populus sp.* Linn. ‘2025’ (also known as Zhonglin 2025, L2025 for short). The leaf color of JHY gradually changes from bright red, during the germination period, to orange-red during the mature period, and the deciduous period to orange-red. The color is bright, varied, and attractive, giving a favorable visual impact over the entire growth period (http://www.zhhyy.net). The RGB color model is a color standard, which produces a variety of colors by changing red (R), green (G), and blue (B) color channels and superimposing them on each other. According to the RGB color system, the superposition color of red and yellow is orange (golden)^[28]^.

In this study, we compared the golden mutant (JHY) to the normal green leaves of poplar variety L2025 in terms of phenotype, pigment content, transcriptome and metabolome. We identified differentially expressed genes (DEGs) related to pigment biosynthesis by transcriptomics, and identified differentially expressed metabolites (DEMs) related to anthocyanin synthesis by metabolomics. According to these results, a model of golden leaf formation is established. Our results are based on changes in physiology, transcription, and metabolism that reveal the underlying mechanism of producing golden leaf color, which provides a reference for the study of tree leaf color.

## RESULTS

### Chlorophyll, carotenoid and Mg^2+^ levels in leaves

**Figure 1:**
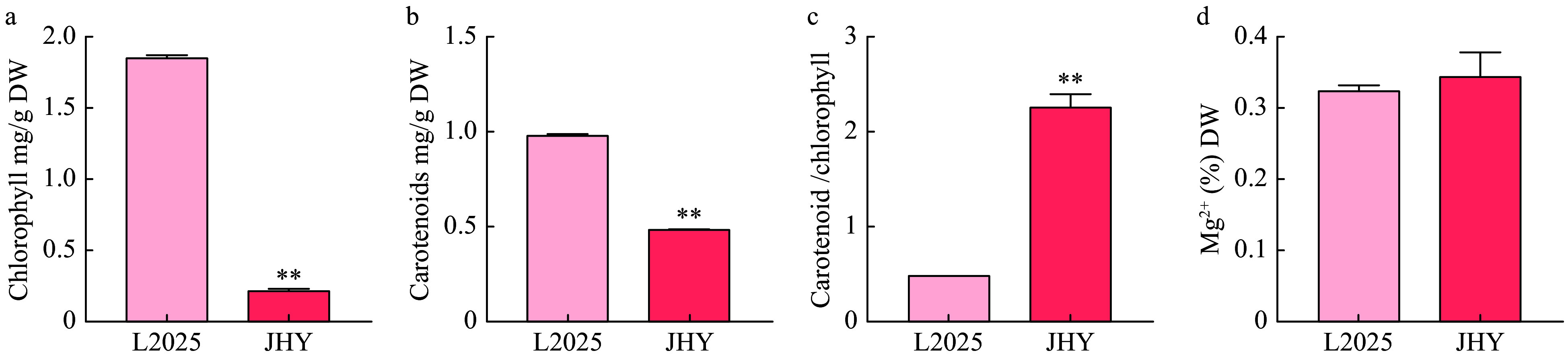
Chlorophyll, carotenoid and Mg^2+^ content levels in leaves of L2025 and JHY. (a) Total chlorophyll content in leaves of two varieties; (b) Total carotenoid content in leaves of two varieties; (c) The carotenoid and chlorophyll content ratio in leaves of two varieties; (d) Total Mg^2+^ content in the leaves of the two varieties.

The yellowing of green leaves is affected by many factors, one such factor being the content of pigments. Therefore, the content of chlorophylls and carotenoids in L2025 and JHY were determined. The Mg^2+^ content was also determined as Mg^2+^ is an important component of the chlorophyll molecule. The chlorophyll content was 1.85 mg/g DW (dry weight) in L2025, but only 0.21 mg/g DW in JHY. The total chlorophyll content in L2025 was about 10 times that of JHY (Fig. 1a). This result shows that the yellow leaf trait is closely related to decreased chlorophyll content. Similar to the large chlorophyll content difference, carotenoid content also differed in the two poplar varieties (Fig. 1b), the difference was however less pronounced than the chlorophyll content. The content of carotenoids in L2025 was 0.29 mg/g DW and the content in JHY was 0.08 mg/g DW. The contents of carotenoids in JHY were about one third of that in L2025. The carotenoid and chlorophyll ratio were however much higher in JHY (Fig. 1c). We further measured the content of Mg^2+^ in two varieties and the results indicated L2025 and JHY had no significant difference (Fig. 1d), indicating that the decrease of chlorophyll in JHY was not caused by the absence of Mg^2+^.

### Transcriptomic analysis of L2025 and JHY

To study the molecular basis of color changes in the golden leaves of JHY, library preparation and RNA-seq were performed on L2025 and JHY. A total of 251.602 M raw reads were produced, and after filtering these raw reads, we obtained a total of 233.743 M clean reads: 119.513 M reads and 114.23 M reads were generated for the L2025 and JHY samples, respectively (Supplemental Table S1). The data was submitted to the NCBI database under the SRA number of PRJNA558190 (three replications of L2025) and PRJNA699052 (three replications of JHY). A principal component analysis and Pearson correlation analysis showed that there were highly correlated transcriptome characteristics between the biological replicates of each group of samples (Supplemental Fig. S1a−b). To explore the functions of the unigenes and obtain annotation information for the transcripts, a BLAST search was conducted. Functional annotations were performed on multiple public databases including the National Center for Biotechnology Information (Nr), Kyoto Encyclopedia of Genes and Genomes (KEGG), Gene Ontology (GO), Swiss-Prot, euKaryotic Ortholog Groups (KOG), Protein family (Pfam) and Evolutionary Genealogy of Genes: Non-supervised Orthologous Groups (eggNOG). A total of 53,016 transcripts were distributed over the seven databases as follows: 42,061 in Nr (79.30%), 30,514 in GO (57.60%), 13,637 in KEGG (25.70%), 27,832 in KOG (52.50%), 26,720 in Pfam (50.40%), 21,351 in SwissProt (40.30%), and 41,775 in TrEMBL (78.80%) (Supplemental Fig. S1c). The FPKM distribution of the transcriptome sequence data was visualized in a box-plot to compare the overall transcript expression levels of the different samples. Gene expression between the L2025 and JHY plants was stable (Supplemental Fig. S1d).

### Functional annotation and classification of the DEGs among different poplars

**Figure 2:**
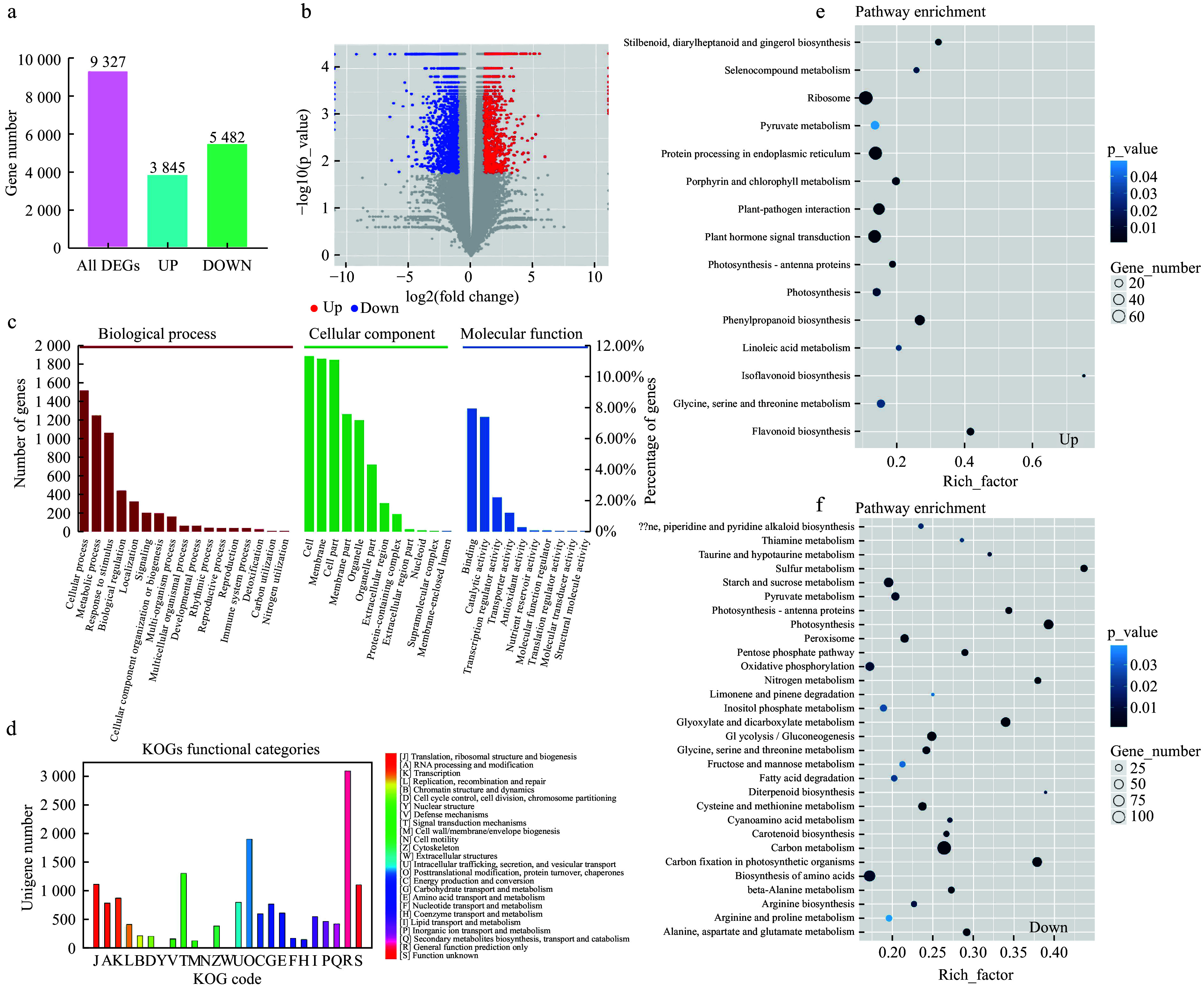
Functional annotation of unigenes in leaf transcriptomes of poplars among different samples. (a) Summary of the transcriptome DEGs. (b) The Volcano Plot of transcriptome DEGs. (c) GO classification of differentially expressed genes. (d) KOG classification of Acer rubrum transcripts. (e) KEGG enrichment of up-regulated genes. (f) KEGG enrichment of down-regulated genes

We detected 9,327 differentially expressed genes (DEGs) between L2020 and JHY, with 3,842 being up-regulated and 5,482 being down-regulated (Fig. 2a−b). We analyzed the GO, KOG, and KEGG pathways to determine the biological functions of DEGs. The GO annotation system consists of three major branches: biological process, molecular function, and cellular component. These unigenes were further divided into 39 major functional terms. Of all the GO categories, cellular process, cell part, and binding were the most over-represented terms in the three GO categories mentioned above, respectively. Nitrogen utilization, membrane-enclosed lumen and structural molecule activity were the least frequent (Fig. 2c). The unigenes enriched by KOG could be assigned to 25 groups. Group R (general function prediction only) was the most highly represented. Group T (signal transduction mechanisms) and O (post-translational modification, protein turnover, chaperones) also shared a high-percentage of genes. For group Y (nuclear structure), group W (extracellular structures) and group N (cell motility), only a few genes were assigned (Fig. 2d). In the KEGG signal enrichment pathway, the most enriched KEGG pathways were ribosome, protein processing in endoplasmic reticulum and carbon metabolism (Fig. 2e−f). As an important secondary metabolite, flavonoid biosynthesis shared the same phenylpropanoid metabolic pathway with plant anthocyanin synthesis. In the L2025 vs JHY comparison, this pathway was significantly enriched. In addition, chlorophyll metabolism and carotenoid metabolism were significantly enriched. A total of 38, 124 and 36 unigenes were involved in anthocyanin biosynthesis, chlorophyll biosynthesis, carotenoid biosynthesis, respectively (Table 1).

**Table 1:** Candidate unigenes involved in anthocyanin biosynthesis, chlorophyll metabolism and carotenoid biosynthesis in JHY.

Function	Gene	Enzyme	Total No.	DEG No.
Phenylpropanoid synthesis pathway	*PAL*	Phenylalanine ammonia-lyase	6	4
	*C4H*	Cinnamate 4-hydroxylase	6	4
	*4CL*	4-Coumarate: CoA ligase	11	5
Flavonoid synthesis pathway	*CHS*	Chalcone synthase	3	2
	*CHI*	Chalcone isomerase	1	1
	*F3H*	Flavanone 3-hydroxylase	2	1
	*F3*’*H*	Flavonoid 3’-hydroxylase	4	2
	*DFR*	Dihydroflavonol 4-reductase	2	1
	*ANS*	Anthocyanidin synthase	1	1
Anthocyanin synthesis pathway	*UFGT*	UDP-glucose: anthocyanidin 3-*O*-glucosyltransferase	1	1
	*3RT*	Anthocyanidin 3-glucoside	0	0
	*UGT75C1*	Cyanidin 3-*O*-rutinoside 5-*O*-glucosyltransferase	1	1
ALA formation	*HEMA*	Glutamyl-tRNA reductase	4	1
	*HEML*	Glutamate-1-semialdehyde 2,1-aminomutase	1	0
Proto ΙΧ formation	*HEMB*	Porphobilinogen synthase	2	1
	*HEMC*	Hydroxymethylbilane synthase	1	1
	*HEMD*	Uroporphyrinogen-III synthase	2	1
	*HEME*	Uroporphyrinogen decarboxylase	2	1
	*HEMF*	Coproporphyrinogen-III oxidase	2	0
	*HEMN*	Oxygen-independent coproporphyrinogen-III oxidase	2	0
	*HEMY*	Oxygen-dependent protoporphyrinogen oxidase	8	2
Chlorophyll formation	*CHLH*	Magnesium chelatase subunit H	6	5
	*CHLE*	Magnesium-protoporphyrin IX monomethyl ester (oxidative) cyclase	3	0
	*CHLM*	Magnesium protoporphyrin IX methyltransferase	2	0
	*DVR*	Divinyl chlorophyllide a 8-vinyl-reductase	1	0
	*POR*	Protochlorophyllide reductase	2	1
Chlorophyll cycle	*CAO*	Chlorophyllide a oxygenase	2	0
	*CHLG*	Chlorophyll synthase	1	0
	*CLH*	Chlorophyllase	2	1
	*NYC1*	Chlorophyll (ide) b reductase	1	0
	*HCAR*	7-Hydroxymethyl chlorophyll a reductase	1	0
Chlorophyll degradation	*PAO*	Pheophorbide a oxygenase	6	2
	*RCCR*	Red chlorophyll catabolite reductase	1	1
Carotenes formation	*PSY*	Phytoene synthase	7	1
	*PDS*	Phytoene desaturase	1	0
	*ZISO*	*ζ*-carotene isomerase	2	1
	*ZDS*	*ζ*- carotene desaturase	3	0
	*crtISO*	Carotenoid isomerase	1	1
	*Lcy E*	*ε*-cyclase	2	0
	*Lcy B*	*β*-cyclase	1	1
Xanthophll formation	*LUT5*	*β*-hydroxylase	1	0
	*CCS1*	Capsanthin/Capsorubin synthase	2	1
	*LUT1*	*ε*-cyclase	1	0
	*VDE*	Violaxanthin de-epoxidase	1	0
	*ZEP*	Zeaxanthin epoxidase	4	3
	*NXS*	Neoxanthin synthase	0	0
Carotenoid degradation	*NCED*	9-*cis*-Epoxycarotenoid dioxygenase	7	2
	*AAO3*	Abscisic-aldehyde oxidase	3	0

### The Senescence-associated genes (SAGs) did not show up -regulation in JHY

Leaf senescence in autumn can also lead to leaf yellowing. These senescence-associated genes (SAGs) are marker genes of leaf senescence, and the expression of *SAGs* in the leaves shows the aging degree of the leaves. In our data, we found 14 *SAGs*, all of which were slightly down-regulated. This result indicates that the yellowing of JHY leaves is not caused by leaf senescence (Supplemental Fig. S2a).

### Differential expression of chlorophyll synthesis genes

**Figure 3:**
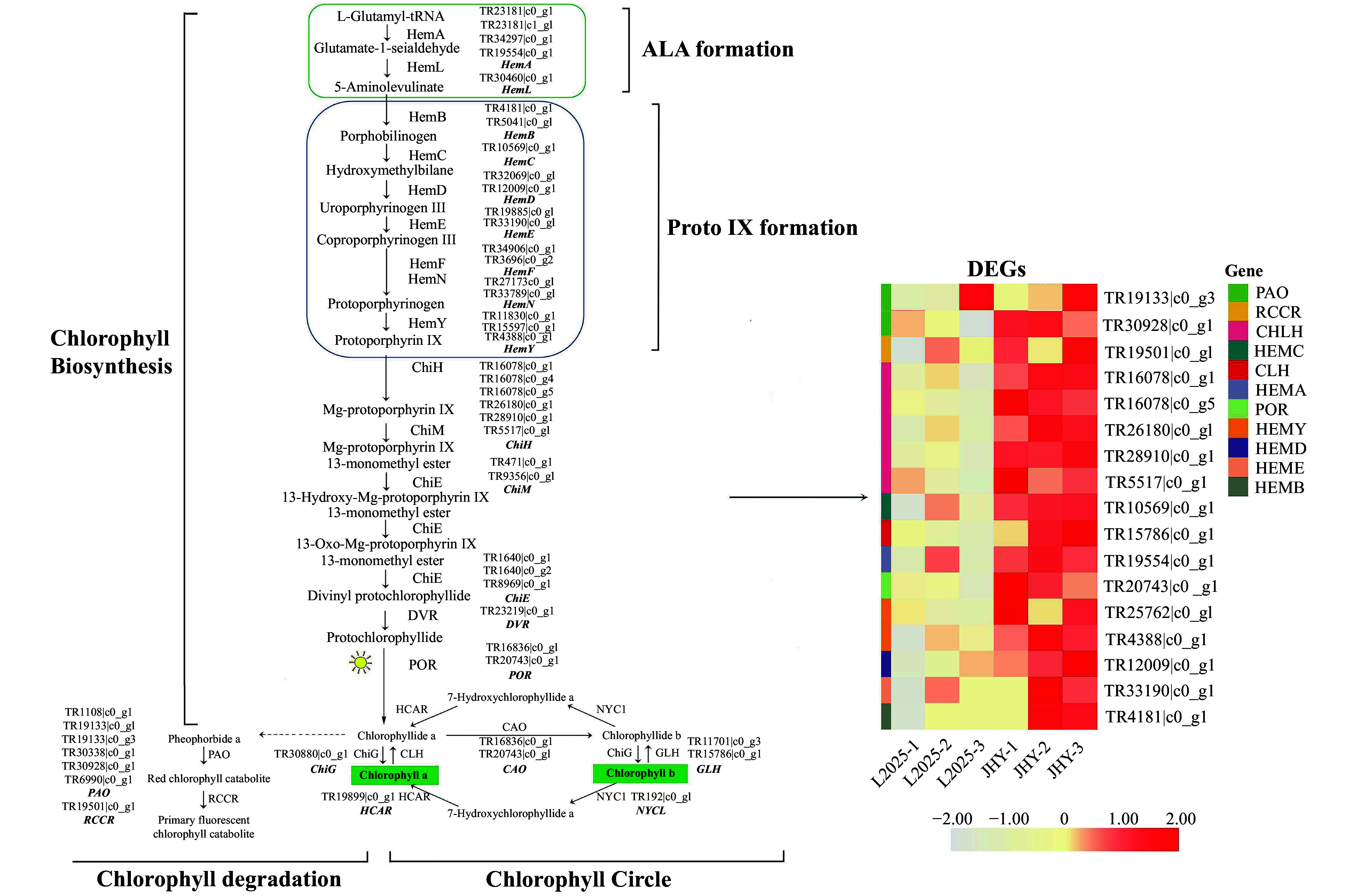
Differential expression of unigenes related to the chlorophyll metabolism pathway. The expression level was based on FPKM value. The darker the color, the higher the gene expression level.

The chlorophyll metabolic pathway in higher plants involves three stages. Changes in any of these three stages will result in chlorosis of leaves. Therefore, we focused on the core genes encoding enzymes involved in chlorophyll metabolic pathways. We identified 116 candidate genes that encoded 24 enzymes related to chlorophyll metabolism. Among them, 17 DEGs with 14 and 3 related to chlorophyll biosynthesis and chlorophyll degradation, respectively (Table 1). HCA (hierarchical cluster analysis) was used to represent different expression levels of DEGs (Fig. 3). Genes in chlorophyll biosynthesis that were significantly up-regulated, included *HemA* (Glutamyl-tRNA reductase), *HemB* (Porphobilinogen synthase), *HemE* (Uroporphyrinogen decarboxylase), *HemC* (Hydroxymethylbilane synthase) and *POR* (Protochlorophyllide reductase). The process of protoporphyrin ΙΧ to Mg-protoporphyrin ΙΧ is catalyzed by ChlH (Magnesium chelatase subunit H), and the gene expression of *ChlH* was up-regulated. In addition, two important enzymes in the chlorophyll degradation process are PAO (Pheophorbide a oxygenase) and RCCR (Red chlorophyll catabolite reductase), and the genes regulating these two enzymes were also significantly up-regulated. The transformation of chlorophyll-b to chlorophyll-a is the primary route for the degradation of chlorophyll-b^[29]^. CLH (Chlorophyllase) plays an important role in the chlorophyll cycle and chlorophyll degradation. Degradation of chlorophyll starts from the dissociation of the chlorophyll-protein complex. Initially, under the catalysis of CLH, chlorophyll-a produces chlorophyllide-a. Chlorophyllide-a undergoes a series of processes to degrade chlorophyll into non-fluorescent products^[30, 31]^. In our data, *CLH* was up-regulated in JHY. This result showed that although the chlorophyll synthesis gene was significantly up-regulated, the chlorophyll degradation gene was also significantly up-regulated. The interaction of the two genes may be a major factor causing the decrease of chlorophyll content. In addition, the photosynthesis-related genes in the leaves are up-regulated, which further proves that the yellowing of the leaf color of JHY is not caused by leaf senescence (Supplemental Fig. S2b).

### Down-regulation of DEGs in the carotenoid metabolism pathway

**Figure 4:**
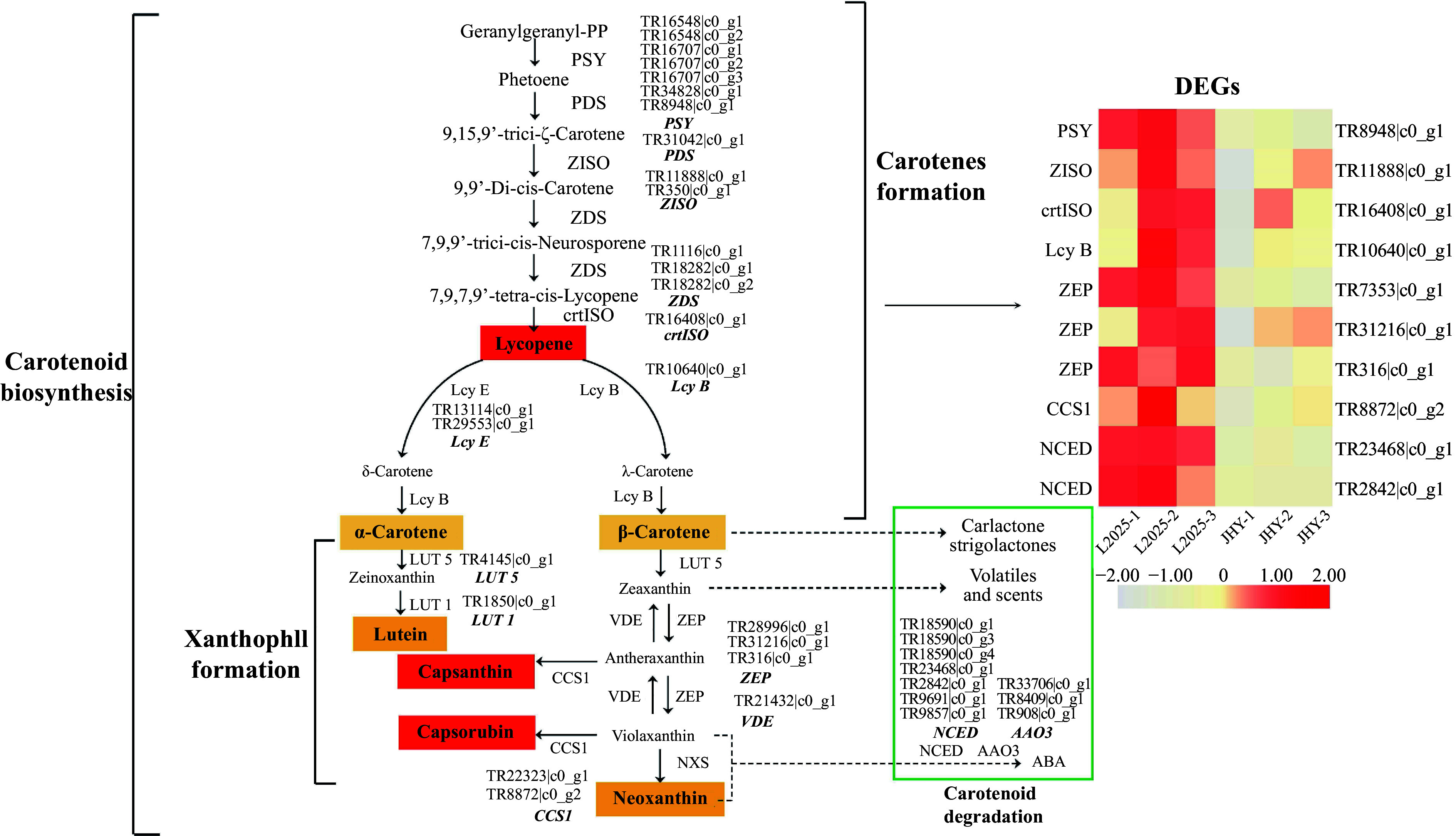
Differential expression of the unigenes related to carotenoid metabolism pathways. (The expression level was based on FPKM value. The darker the color, the higher the gene expression level.)

Carotenoids are involved in the growth and development of plants and are pigments affecting plant color. The photosynthetic system in chloroplast I and II protein complexes contains carotenoids, and their effect is on quenching excess light energy. Carotenoids with higher levels in the chloroplast include *β*-carotene and lutein^[14]^. We identified 36 candidate genes that encoded 16 enzymes related to carotenoid metabolism and 10 of them were DEGs (Fig. 4, Table 1). The genes related to carotene synthesis, such as *PSY* (Phytoene synthase), *ZISO* (*ζ*-carotene isomerase), *crtISO* (Carotenoid isomerase), and *lcy B* (*β*-cyclase) were down-regulated, and this would reduce the amount of carotene. ZEP (Zeaxanthin epoxidase) is an important enzyme in the xanthophyll cycle, and it can catalyze the conversion of zeaxanthin from violaxanthin to antheraxanthin^[32]^. This process is also the key to ABA biosynthesis^[33]^. The genes that regulate ZEP enzymes were down-regulated. Down-regulation of the expression levels of these genes reduced in xanthophyll. ABA synthesis is mainly achieved through the oxidative cleavage of carotenoids, and NCED (9-*cis*-epoxy carotenoid dioxygenase) is a key enzyme in ABA biosynthesis^[34]^. During the conversion of carotene to ABA, *NCED* is also down-regulated. This result may lead to reduced abscisic acid content in JHY. All of the DEGs involved in carotenoid metabolism were down-regulated, which is consistent with the decrease in carotenoid content. This provided further evidence that carotenoids have little effect on the yellowing of JHY.

### Up-regulation of DEGs and DEMs in the anthocyanin metabolism pathway

**Figure 5 Figure5:**
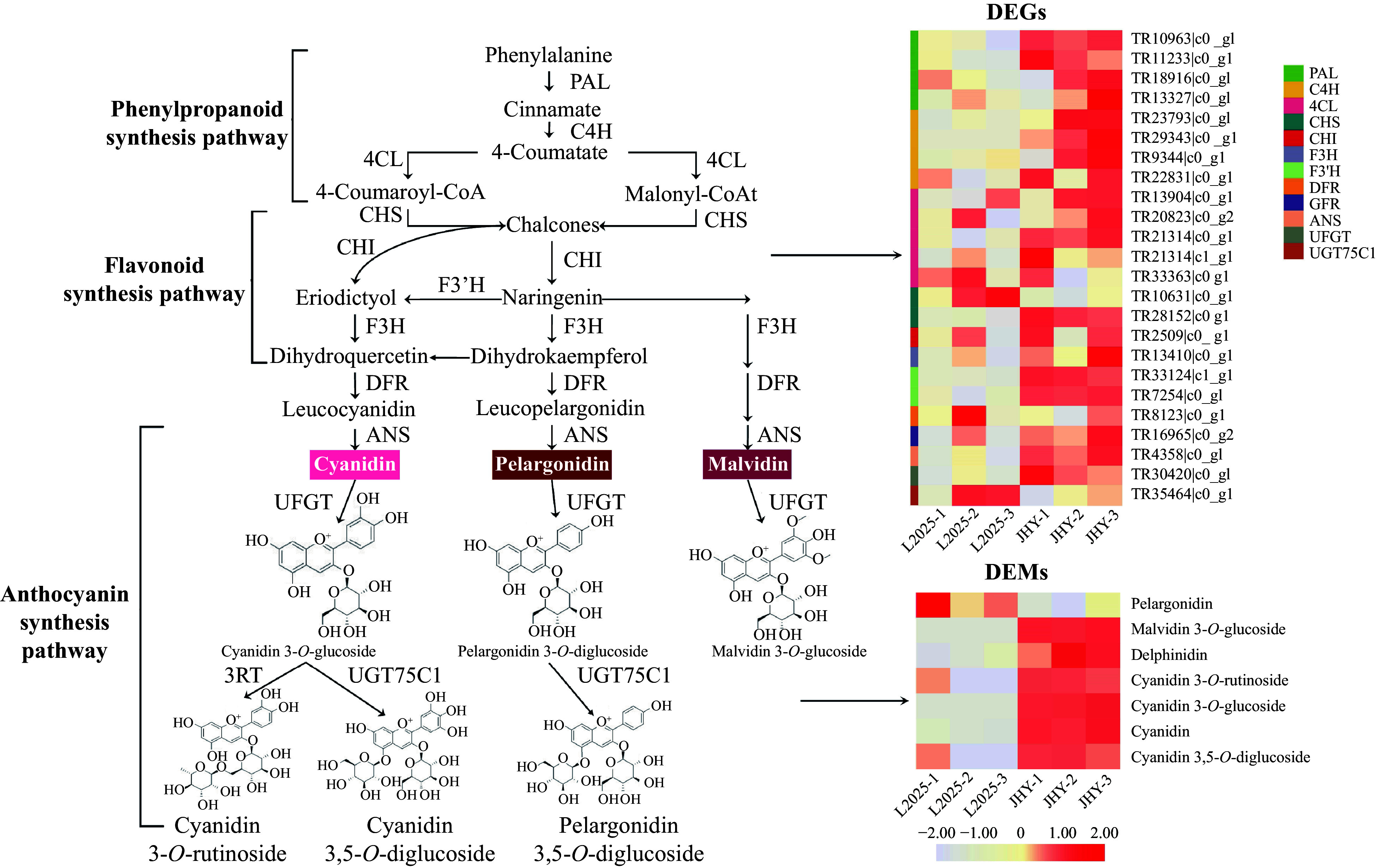
Differential expression of unigenes and metabolites related to the carotenoid metabolism pathway. (The expression level was based on FPKM value. The darker the color, the higher the gene expression level.)

Our data showed significant up-regulation of anthocyanin synthesis gene expression and anthocyanin metabolite accumulation. In addition to a number of gene expression changes in chlorophyll and carotenoid metabolic pathways, significant changes in gene expression were also observed in the genes regulating anthocyanin accumulation. We identified 24 DEGs involved in the anthocyanin metabolic pathway. With the exception of *DFR* (Dihydroflavonol 4-reductase) and *UFGT7C1* (Cyanidin 3-*O*-rutinoside 5-*O*-glucosyltransferase), all of them were significantly up-regulated (Fig. 5, Table 1). Therefore, the leaf color changes of JHY appear to be closely related to anthocyanin accumulation. To investigate the accumulation of anthocyanins in different poplar varieties, 12 anthocyanins and 4 anthocyanidins were identified using HPLC-ESI-MS/MS. The molecular weight, retention time, and Q1/Q3 pairs, of the 16 anthocyanins and their accumulation in L2025 and JHY are listed in Table 2. We also show the accumulation patterns of several common differential metabolites (DEMs)[Fig Figure5], named cyanidin 3-*O*-glucoside, cyanidin 3-*O*-rutinoside, cyanidin, cyanidin 3,5-*O*-diglucoside, pelargonidin, malvidin 3-*O*-glucoside and delphinidin (Fig. 5). These results showed that anthocyanin accumulation was higher in JHY than in L2025. For example, malvidin 3-*O*-glucoside, malvidin 3-*O*-galactoside and cyanidin 3-*O*-glucoside are absent in L2025, while high levels occurred in JHY. In addition, the peonidin *O*-hexoside, peonidin 3-*O*-glucoside and delphinidin *O*-malonyl-malonylhexoside levels in JHY were about five times higher than those in L2025. The two anthocyanidins, peonidin and delphinidin, in JHY were present at levels about two times higher than the levels in L2025. Other metabolites such as pelargonidin 3-*O*-beta-*D*-glucoside, cyanidin *O*-syringic acid and cyanidin *O*-diacetyl-hexoside-*O*-glyceric acid were 10 times higher in JHY than in L2025, and cyanidin 3-*O*-rutinoside, cyanidin, cyanidin 3,5*-O*-diglucoside were 20 times higher in JHY. In contrast, we found two metabolites, pelargonidin and peonidin 3, 5-*O*-diglucoside, with lower levels in JHY than L2025. The anthocyanin accumulation analysis showed that the metabolite differences are in accordance with DEGs expression, and the formation of golden leaves is highly related to anthocyanin accumulation.

**Table 2:** List of the anthocyanins and anthocyanidins detected in L2025 and JHY. (Note: The number indicates the areas of the peaks obtained for each compound in the MRM analysis. The relative quantitative results compare the different expressions of the same metabolite, but not the different expressions of different metabolites. Q1, parent ions; Q3, characteristic fragments; RT, retention time.)

Compounds	Q1 (Da)	Q3 (Da)	Rt (min)	Molecular Weight (Da)	L2025	JHY
Peonidin *O*-hexoside	463.10	301.00	3.00	463.123	1560000	5710000
Peonidin 3-*O*-glucoside	463.10	301.10	2.94	498.0929	1490000	6143333
Peonidin	301.10	286.00	3.98	301.1	292333	415000
Pelargonidin 3-*O*-beta-*D*-glucoside	433.10	271.00	2.75	433.1	507666	5330000
Pelargonidin	271.00	149.00	3.75	271.24	76133	31900
Malvidin 3-*O*-glucoside	493.20	331.00	2.87	493.2	0	455666
Malvidin 3-*O*-galactoside	493.00	331.00	2.87	493	0	267333
Delphinidin *O*-malonyl-malonylhexoside	637.10	303.40	3.24	637.1	3183	12600
Delphinidin	303.00	229.00	2.90	303.24	144000	399666
Cyanidin *O*-syringic acid	465.10	285.10	2.50	466.1	361666	3426667
Cyanidin *O*-diacetyl-hexoside-*O*-glyceric acid	619.10	285.20	3.20	620.1	17700	128200
Cyanidin 3-*O*-rutinoside	595.00	287.00	2.62	595	22139	492000
Cyanidin 3-*O*-glucoside	449.10	287.30	2.56	449.1	0	502666
Cyanidin	287.00	213.00	3.45	287.24	73166	1019000
Cyanidin 3,5-*O*-diglucoside	611.00	287.00	2.15	611	112672	2560667
Peonidin 3, 5-*O*-diglucoside	625.40	301.00	2.16	625.4	271666	94166

PCA is commonly used for extracting and rationalizing information from the multivariate analyses of biological systems. The two analyzed varieties were included in the PCA plot, indicating the analysis was stable and repeatable. The six samples from the two varieties grouped into two distinct areas of the PCA plot, suggesting that the observed differences in anthocyanin profiles were correlated with leaf color (Supplemental Fig. S3a). The OPLS-DA model involved pair-wise comparisons of the anthocyanin metabolite contents of the samples to evaluate the differences between L2025 and JHY (R2X = 0.897, R2Y = 1, and Q2 = 0.995). The Q2 values exceeded 0.9, demonstrating that these models were stable and reliable (Supplemental Fig. S3b). Alignment verification on OPLS-DA (n = 200) was carried out by performing 200 alignment experiments. In the model verification, the horizontal lines corresponded to the R and Q of the original model, and the red and blue dots represent the R’ and Q’ of the model after Y replacement, respectively. Both R’ and Q’ were smaller than the R and Q of the original model. The corresponding points do not exceed the corresponding line, indicating that the model is meaningful and the differences in anthocyanin metabolism could be screened further (Supplemental Fig. S3c).

### Validation of selected DEGs by qRT-PCR

**Figure 6:**
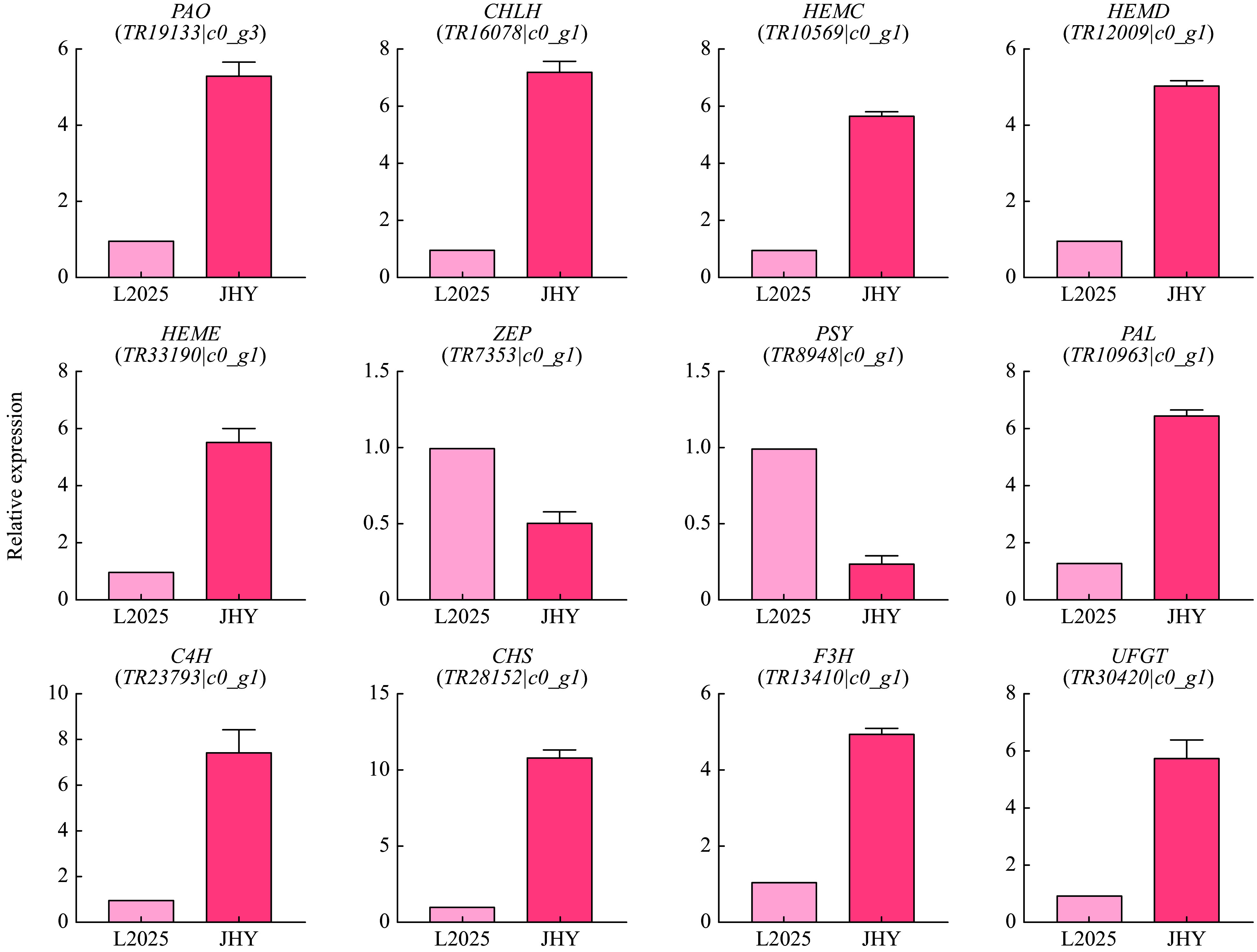
qRT-PCR analysis of the expression of 12 pigmentation-related candidate unigenes in leaves. The Y-axis shows the log_2_ ratio.

We detected the expression of 12 DEGs in L2025 and JHY, including 5 genes involved in chlorophyll metabolic pathway, two genes involved in carotenoid biosynthesis and degradation, and five genes involved in anthocyanin biosynthesis. DEGs (*PAO*, *CHLH*, *HEMC*, *HEMD* and *HEME*) in chlorophyll metabolism were up-regulated, DEGs (*ZEP* and *PSY*) in the carotenoid metabolism pathway were down-regulated, and DEGs (*PAL*, *C4H*, *CHS*, *F3H* and *UFGT*) in the anthocyanin pathway were up-regulated. These results are consistent with the transcription data (Fig. 6) and showed that the transcriptome data are reliable. The meltcurves of qRT-PCR are single-peak and have good repeatability, and the difference of RN value in different cDNA concentration was constant (Supplemental Fig. S4). Primers for qRT-PCR are shown in supplemental files (Supplemental Table S2).

## DISCUSSION

### The accumulation pattern of pigments in Golden Poplar

Leaf color is an important commercial trait of ornamental plants. Golden plant leaves are often caused by an increase in carotenoids^[35]^, our results show however that carotenoid and chlorophyll content exhibits a similar pattern, a significant decrease. Our results are consistent with a previous study on maple trees^[5]^, which showed that the formation of golden plants is influenced by a variety of pigments including anthocyanins. The anthocyanin content in JHY, determined by HPLC-ESI-MS/MS, indicated that the content of most anthocyanins increased significantly. These also provides favorable conditions for the appearance of golden color in the leaves. In addition, the ratio difference between chlorophyll content and carotenoid content is also one of the important reasons causing leaf color change. The differences between the levels of these three kinds of pigments in JHY and L2025 results in the golden leaf trait of JHY.

### Do yellowing leaves affect plant growth and the aging process?

As we all know, chlorophyll is an indispensable pigment for photosynthesis in plants. The content of chlorophyll also affects the growth and development of plants. A variety of studies have shown that the senescence of leaves is mainly due to the degradation of chlorophyll, which leads to the appearance of carotenoid color and results in the the leaves turning yellow^[36, 37]^. In previous reports, chlorophyll content decreased in senescent leaves of poplars, accompanied by reduced photosynthetic efficiency and down-regulation of a large number of genes associated with photosynthesis^[38, 39]^. Here, we found the content of chlorophyll in JHY leaves was significantly lower than L2025, most of the photosynthesis related gene was significantly up-regulated. We suggested that although the reduction of chlorophyll content inhibits plant growth and development, that the up-regulation of genes related to photosynthesis may cancel this out to some degree^[40]^. We then investigated whether the yellowing of leaves is caused by senescence as golden leaves seen in autumn are mostly due to aging^[41]^. SAGs are the marker genes of leaf senescence^[42]^. In our study, the down-regulation of SAGs in JHY indicates that the signs of leaf senescence are weak and the yellowing of leaves will not affect the aging process in JHY.

In addition, NCED is down-regulated in JHY, and NECDs are rate-limiting enzymes that control the conversion of carotenoids to ABA, mainly by catalyzing the cleavage of violaxanthin or neoxanthin to form ABA precursor^[43]^. ABA is an important indicator for studying plant growth and stress resistance. This result provides a basis for subsequent research on ABA content and the corresponding response performance of JHY.

### Prediction model of golden leaf formation based on RGB

**Figure 7:**
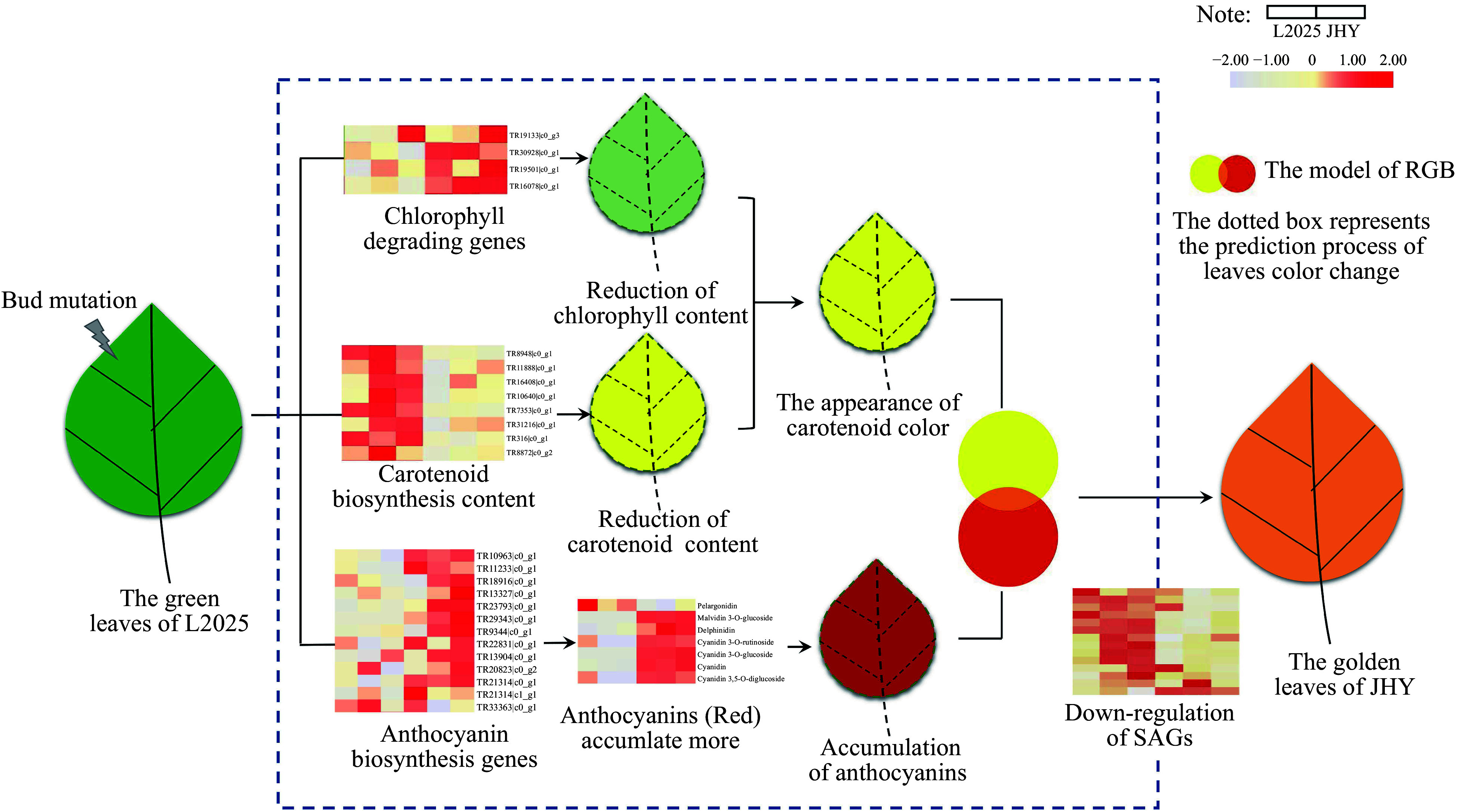
The regulation pattern of golden leaf formation.

The chlorophyll content in JHY was much lower than that in L2025, and this could be as a result of the genes regulating chlorophyll degradation also being up-regulated. PAO encodes a monooxygenase that catalyzes the oxidation of pheophytin a PAO encodes a monooxygenase that catalyzes the oxidation of pheophytin a, which provides the basis for further decomposition and is an important factor in yellowing leaves^[44−48]^. Although the expression of the chlorophyll synthesis gene was up-regulated, the high expression of *PAO* still led to a greater decrease in chlorophyll content in JHY. Similar to the trend of chlorophyll change, the level of carotenoid also decreased. PSY is the primary rate-limiting enzyme in the carotenoid synthesis pathway, and is also an extensively studied carotenoid metabolic enzyme^[49]^. The FPKM value of *PSY* in L2025 is higher than that in JHY, which directly led to the decrease of carotenoid content. The two ends of lycopene are catalyzed by Lcy B and Lcy E (*ε*-cyclase) to form α/β-carotene^[50]^. *Z-ISO*, *crt-ISO* and *Lcy B* were significantly down-regulated at this stage, resulting in lower carotenoid levels. Although the content of chlorophyll and carotenoid are both down-regulated, the decline of carotenoid is less than that of chlorophyll (Fig. 2), which indicates that carotenoid color (yellow) may be more dominant. UFGT (UDP-glucose: anthocyanidin 3-*O*-glucosyltransferase) is the last enzyme encoded by structural genes in the anthocyanin synthesis pathway. It catalyzes the glycosylation of unstable anthocyanin to form a stable anthocyanin. This is a key process to ensure the stability and water solubility of anthocyanin^[51]^. The high expression of UFGT may be one reason for the high levels of Peonidin 3-*O*-glucoside, Malvidin 3-*O*-glucoside and Cyanidin 3-*O*-glucoside in JHY. Malvidin 3-*O*-glucoside, Cyanidin 3-*O*-glucoside and many up-regulated anthocyanins are all reddish pigments. As mentioned previously, a variety of colors can be superimposed by the three colors of red, green and blue, which is recognized as the RGB system. The superimposed color of red and yellow is orange (golden). Therefore, the yellow color of carotenoids and the red color of anthocyanins can be superimposed resulting in golden leaves, which is the color of JHY leaves (Fig. 7). In addition, Cyanidin *O*-syringic acid was first reported in *Camellia sinensis*^[52]^, and here, we reported its existance in poplars.

## CONCLUSIONS

In summary, through the determination of phenotype and pigment content, we found that the chlorophyll and carotenoid content of JHY are lower than L2025, but the ratio of carotenoid/ chlorophyll is higher than L2025. The difference between the two pigments is one of the reasons for the change in leaf color. At the metabolic level, the anthocyanin content in JHY is significantly higher than that in L2025, which also validates the significant upregulation of the anthocyanin synthesis gene in JHY. Thus, the superposition of chlorophyll, carotenoid and anthocyanin colors contributes to the formation of golden leaves. Furthermore, genes involved in ABA synthesis were significantly up-regulated in JHY, and if there is a correlation between plant leaf color changes and stress resistance will be the focus of our future research.

## MATERIALS AND METHODS

### Plant materials

**Figure 8:**
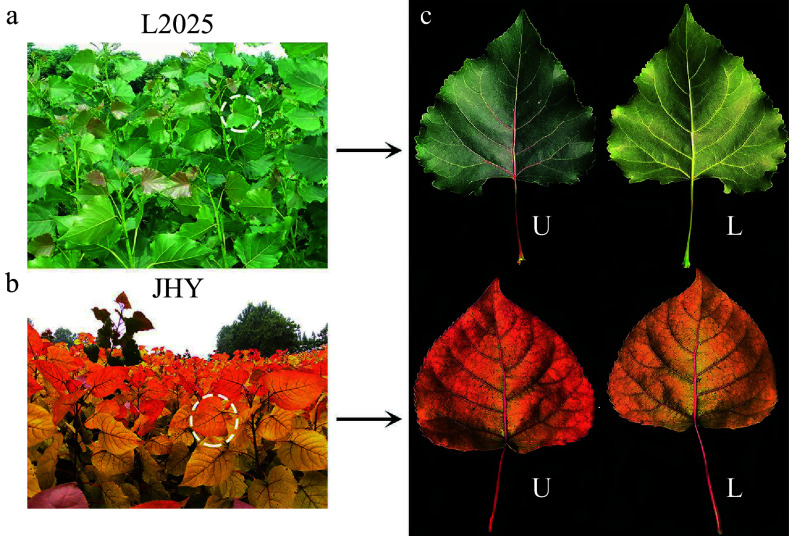
The phenotypes of L2025 and JHY. (a−b) Phenotypes of L2025 and JHY seedlings. (c) The upper (U) and lower (L) epidermis of the second to the fifth fully expanded mature leaves

The poplar variety ‘Zhonglin 2025’ (L2025) was provided by the Chinese Academy of Forestry (Beijing, China). JHY was obtained from the Zhongxing Seedling Planting company (Henan, China). For each variety, five seedlings were planted in pots (15 L capacity) filled with a commercial growth medium comprising perlite, vermiculite, and peat in the spring of 2017. The soil moisture content in the pots was maintained at 70 % measured by a TRIME-PICO 64/32 TDR portable soil moisture meter (IMKO, Germany). The growing conditions for the seedlings in the pots were consistent with the climatic conditions of Shangqiu (city) in Henan Province. Henan province has a warm temperate continental monsoon climate. The annual average temperature is 14 °C, with 726.5 mm annual precipitation and a 271-day frost-free period. The average daylight hours in the four seasons are 620.8 h, 599.8 h, 407.7 h and 376.7 h, and the average temperatures are 16.1 °C, 26.8 °C, 15.3 °C and 3.6 °C, respectively. In September 2018, 12 pots with uniformly growing plants of each variety were selected for subsequent analyses. From 9:00 a.m. to 9:30 a.m., the second to the fifth fully expanded leaves in the mature period (The leaves are orange-red and golden) were harvested from the 1.5-year-old plants (Fig. 8a−c), evenly mixed and immediately frozen with liquid nitrogen. The samples were then stored at −80 °C and used for content determination, metabolite detection, RNA-sequencing and qRT-PCR analysis.

### Determination of chlorophyll, carotenoid and Mg ^2+^ content

Chlorophyll and carotenoids were extracted from freeze-dried leaves with 95% ethanol, after which the chlorophyll content was measured as previously described^[53]^. The absorbance of the extracts was determined at 470, 645, and 663 nm using a spectrophotometer. The protocol for the determination of Mg^2+^ content: Dry weights (DWs) of the samples were determined after oven drying at 80 °C for 72 h. The dried samples were then ground to powder in mortars. Before the Mg^2+^ concentrations were confirmed, the powdered samples were dissolved by adding 5 mL of concentrated sulfuric acid and 2 mL of 30% H_2_O_2_ on an anti-boiling furnace at 350 °C until the liquid became colorless. An atomic absorption spectrophotometer was used to measure the Mg^2+^ content^[54]^. Three replicate measurements were made per treatment.

### Transcriptome analysis

Total RNA was isolated and purified using the CTAB method^[55]^. The integrity, purity, and concentration of the purified RNA were assessed with the Agilent 2100 Bioanalyzer and the NanoDrop ND-1000 spectrophotometer (NanoDrop Technologies, Wilmington, DE, USA). The mRNA extracted from the total RNA in the samples was isolated using Oligo dT. Libraries, were generated and purified using the NEBNext® Ultra™ RNA Library Prep Kit for Illumina® (New England Biolabs Inc., Ipswich, MA, USA) and AMPure XP Beads (Beckman Coulter, Inc., Indianapolis, IN, USA), using the fragmented mRNA as the template, following the manufacturer’s recommendations. The concentration, integrity, and quantification of the library were determined using a Qubit™ Fluorometer (Thermo Fisher Scientific, Waltham, MA, USA), the KAPA Library Quantification Kit (KAPA Biosystems, Wilmington, MA, USA), and a Qsep100 DNA Analyzer (KAPA Biosystems), respectively. The denatured libraries were subject to high-throughput parallel sequencing of both ends of the library using an Illumina HiSeq X™ Ten System sequencing platform. The quality of the raw data was evaluated using FasQC v0.10.1 (http://www.bioinformatics.bbsrc.ac.uk/projects/fastqc/) with default settings. The clean data were separated using Cutadapt v1.9 (http://cutadapt.readthedocs.org/) and the quality threshold was set to Q30, which removed the sequencing adapters and the primer sequence from the raw data to filter out low-quality data. In this study, de novo transcriptome assembly was performed according to Grabherr^[56]^. The transcript level was quantified using Cufflinks (version 2.2.1), and the length of the transcript in the sample was normalized to fragments per kilobase of exon per million reads mapped (FPKM) values^[57]^. The false discovery rate was used to adjust the *P* values of differentially expressed genes (DEGs). Genes with an expression-level change of log_2_ > 2 and an adjusted *P* value < 0.05 were considered DEGs, and were further annotated based on gene ontology (GO) terms and Kyoto Encyclopedia of Genes and Genomes (KEGG) pathways. The enrichment of specific KEGG pathways among the DEGs was assessed with Fisher’s exact test.

### Measurement of Anthocyanins Metabolites by HPLC-ESI-MS/MS

Freeze-dried leaf samples were crushed with a MM 400 mixer mill (Retsch) and zirconia beads for 1.5 min at 30 Hz. The crushed samples were weighed, after which 100 mg powder was mixed with 1.0 mL 70% aqueous methanol for an overnight extraction at 4 °C. After centrifuging the solutions at 10,000 × g for 10 min, the extracts were added to a CNWBOND Carbon-GCB SPE Cartridge (250 mg, 3 mL) and then filtered through a SCAA-104 membrane (0.22 μm pore size; (ANPEL, Shanghai, China) for liquid chromatography-mass spectrometry (LC-MS) analysis.

For each variety, three biological replicates were independently and randomly analyzed to minimize the analysis bias. Sample extracts were analyzed with an HPLC-electrospray ionization (ESI)-tandem mass spectrometry (MS/MS) system (HPLC, Shim-pack UFLC SHIMADZU CBM30A system; MS/MS, Applied Biosystems 6500 Q TRAP), with the following conditions: HPLC: column, Waters ACQUITY UPLC HSS T3 C18 (1.8 µm, 2.1 mm × 100 mm); solvents, solvent A (water and 0.04% acetic acid) and solvent B (acetonitrile and 0.04% acetic acid); gradient program, 100:0 V(A)/V(B) at 0 min, 5:95 V(A)/V(B) at 11.0 min, 5:95 V(A)/V(B) at 12.0 min, 95:5 V(A)/V(B) at 12.1 min, and 95:5 V(A)/V(B) at 15.0 min; flow rate, 0.40 mL/min; temperature, 40 °C; and injection volume, 2 μL. The eluate was analyzed with an ESI-triple quadrupole (QQQ)-linear ion trap (Q TRAP) mass spectrometer. The linear ion trap (LIT) and QQQ scans were acquired with the API 6500 Q TRAP LC-MS/MS system equipped with an ESI Turbo Ion-Spray interface. The ESI source operation parameters were as follows: ion source, turbo spray; source temperature, 500 °C; ion spray voltage, 5,500 V; curtain gas, 25.0 psi; and the collision gas was high. In the QQQ, each ion pair was scanned for detection based on the optimized decompression potential and collision energy^[58]^. The identification and quantification of changes in flavonoids were performed using molecular formula-based mass accuracy and specific features of their MS^2^ spectra.

### Qualitative and quantitative analysis of metabolites

The identification of metabolites detected by the LC-ESI-MS/MS system was performed based on a search of accurate masses of significant peak features against the online MWDB (metware database from Metware Biotechnology Co., Ltd, Wuhan)^[58−60]^. The MWDB was based on the MS2 spectral tag (MS2T) library, which was constructed by Metware Biotechnology Co., Ltd. The annotation of metabolites in the MS2T library was carried out by matching the fragmentation pattern (delivered by ESI-Q TRAP-MS/MS), combined with the retention time and the accurate m/z value (delivered by ESI-QqTOF-MS/MS). Isotope signals, repeated signals of K^+^, Na^+^, and NH_4_^+^, and repeated signals of fragment ions of substances with a relatively high molecular weight were removed in the analysis^[58]^. To produce a maximal signal, collision energy and de-clustering potential were optimized for each precursor–product ion (Q1–Q3) transition.

Metabolites were quantified with the MRM mode of QQQ MS. In this mode, the quadrupole first screened the precursor ions of the target substance and eliminated the ions of other molecular weight substances to prevent preliminary interference. The precursor ions were ionized in the collision chamber and then fragmented. The fragment ions were filtered through the QQQ to obtain a fragment ion with the required characteristics and eliminate the interference of non-target ions. After obtaining the spectral data of metabolites for all samples, the peak area of all MS data was integrated, and the MS peaks of the same metabolite in different samples were corrected by integrating peaks^[61]^. Finally, relative quantitation, which was indicated by the mass spectrum peak area of characteristic ions, was used to measure the content of metabolites.

### Sample quality control analysis

Quality control samples, which were prepared by combining all of the sample extracts, were analyzed using the same method as for the experimental samples. The quality control samples were injected after every five experimental samples throughout the analytical run to assess the repeatability and reliability of the data.

### Validation of DEGs through qRT-PCR

Total RNA extracted from the leaves of L2025 and JHY was used for reverse transcription with the FastQuant RT Kit with DNase (TianGen Biotech Co., Ltd., China) to synthesize the first-strand cDNA. A qRT-PCR assay was performed with an optical 96-well reaction plate, the ABI PRISM 7500 Real-time PCR system (Applied Biosystems), and SuperReal PreMix Plus SYBR Green (TianGen Biotech Co., Ltd). Each reaction contained 12.5 µL SYBR Premix ExTaq, 0.5 µL ROX Reference Dye, 2.0 µL cDNA, and 1.0 µL gene-specific primers in a final volume of 25 µL. All the primer concentrations are 10 μΜ. The PCR program was as follows: 95 °C for 10 s and then 45 cycles at 95 °C for 5 s and 60 °C for 40 s. The qRT-PCR data were analyzed according to the 2^−∆∆*C*T^ method^[62]^. The RNA concentration ranged from 600 ng/ul to 800 ng/ul, and the A260/A280 value ranged from 1.8 to 2.0. The initial concentration of cDNA was 1 ug/ul. The cDNA concentration was diluted according to four gradients, which were 10^0^, 10^−1^, 10^−2^, 10^−3^, respectively. The E value (amplification efficiency) of the qRT-PCR was between 90% and 110%, and R^2^ was greater than 0.99. The qRT-PCR of each gene was carried out for three experimental repetitions, and each experiment was using three biological repetitions^[63]^.

### Statistical analysis

The metabolome and transcriptome analyses were completed with three biological replicates. The PCA and OPLS-DA were completed with R (http://www.r-project.org). The criteria of fold-change ≥ 2 or ≤ 0.5 and a VIP (variable importance in projection) score > 1 were used for identifying differentially accumulated metabolites among the poplar varieties analyzed^[64]^. The content of all differentially accumulated materials or gene expression was analyzed by hierarchical cluster analysis based on the Multi Experiment Viewer (MEV) 4.9.0 software. Computerized algorithms were used by the maximum difference normalization method to homogenize the metabolites or transcript expression levels in MeV^[65]^. Data were analyzed with SPSS 23.0 program software (** represents *p* < 0.01, * represents *p* < 0.05). The figures presented herein were drawn with the GraphPad Prism 8 program and Adobe Illustrator CC.

## SUPPLEMENTARY DATA

Supplementary data to this article can be found online.
