# Supplementary material for: The coloring mechanism of a novel golden variety in Populus deltoides based on the RGB color mode
Source: For Res (Fayettev). 2021 Feb 22;1:5. doi: 10.48130/FR-2021-0005 (PMC11524229; doi:10.48130/FR-2021-0005)

The meltcurve of CHLH

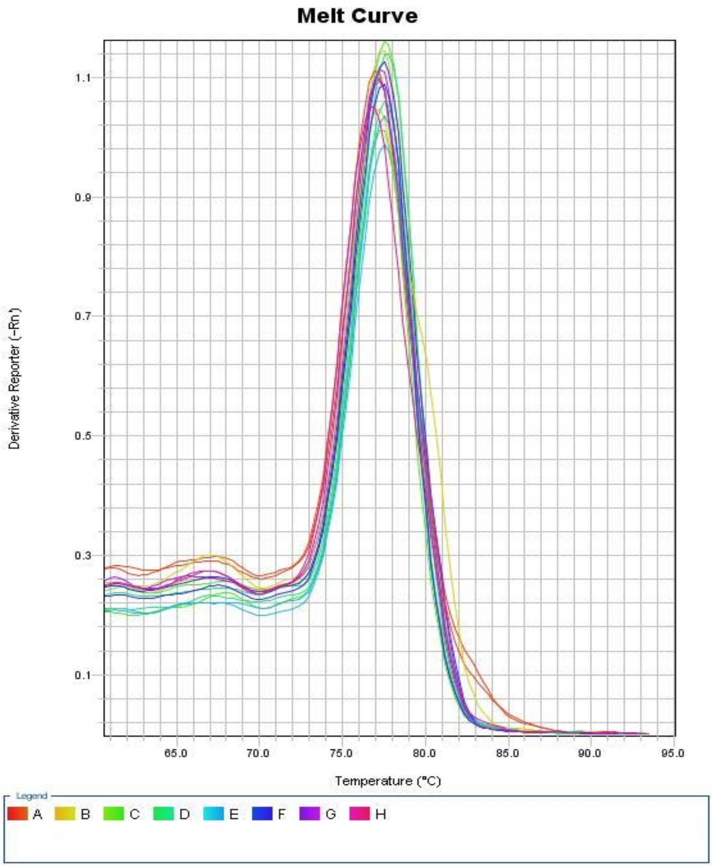


The amplification plot of CHLH

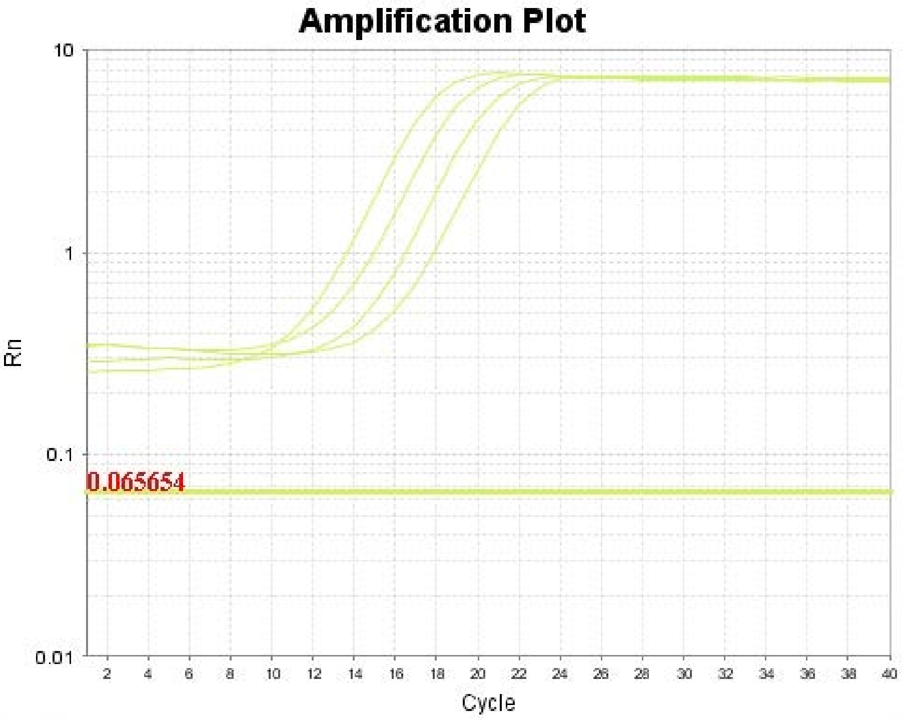

The meltcurve of PSY

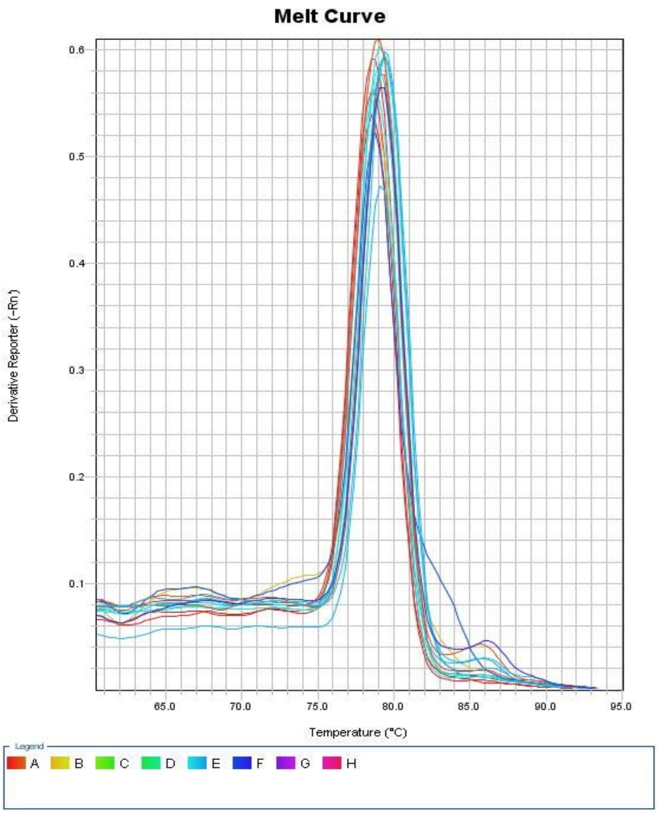


The amplification plot of PSY

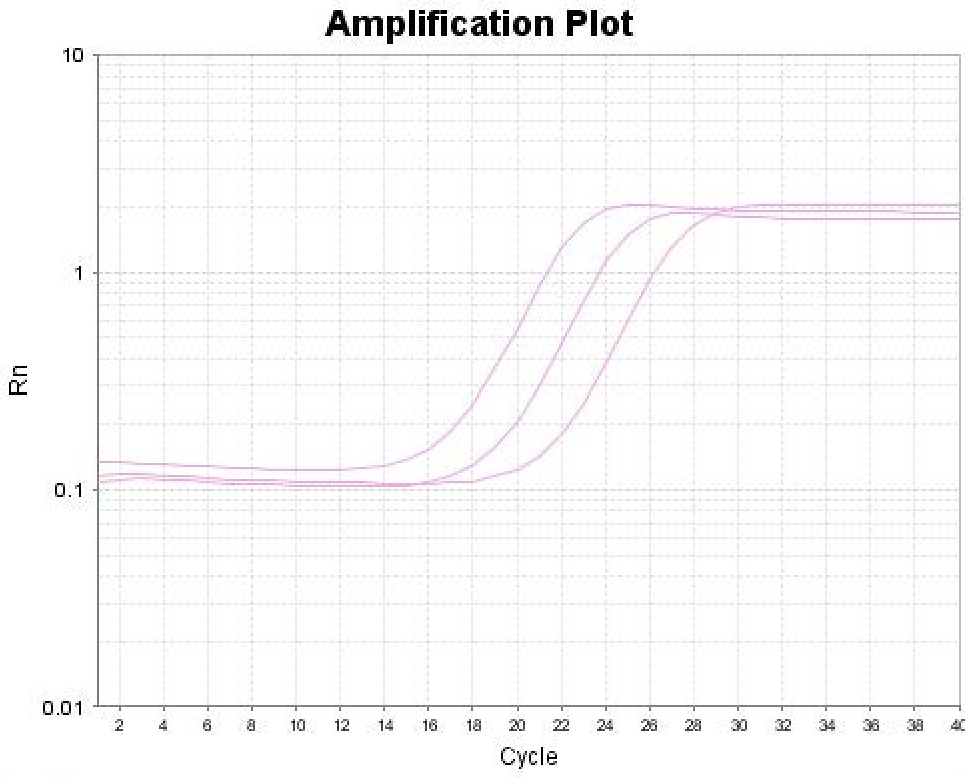

The meltcurve of PAL

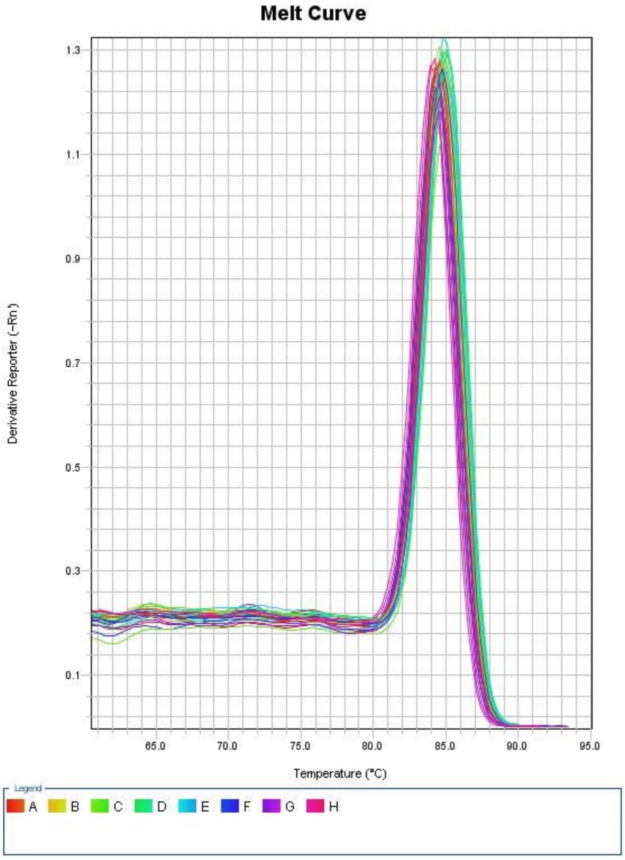


The amplification plot of PAL

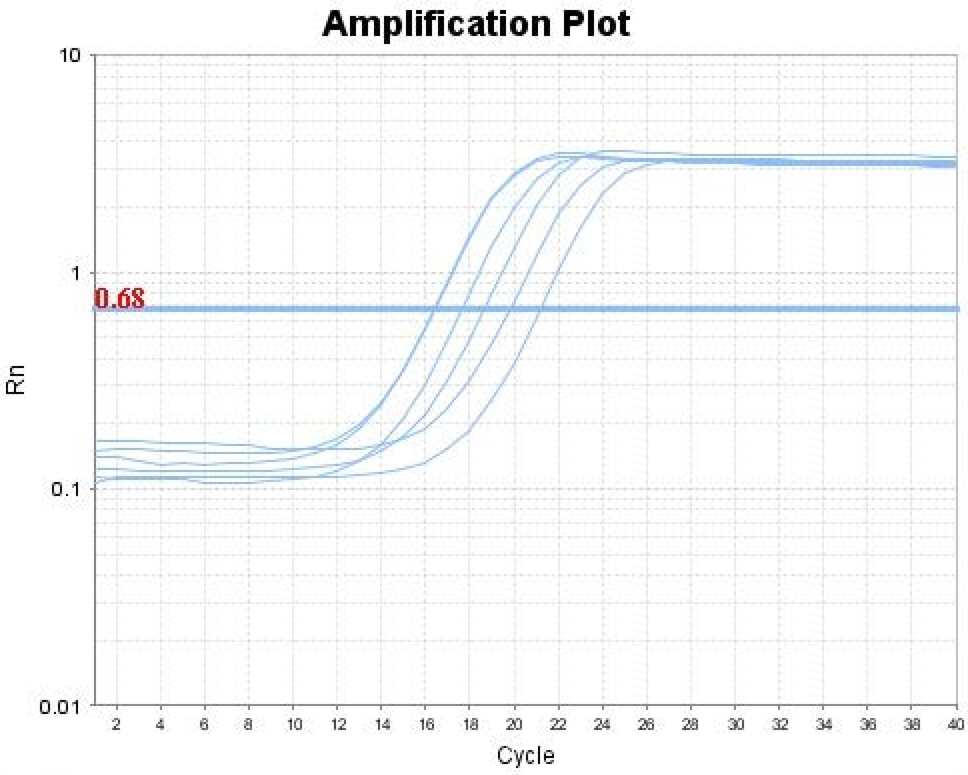

Supplement: Supplementary file 1 — Supplementary data to this article can be found online. [file FR-2021-0005-S1.zip › 10.48130_FR-2021-0005-Suppl-FigureS4.pdf]
