# Supplementary material for: The coloring mechanism of a novel golden variety in Populus deltoides based on the RGB color mode
Source: For Res (Fayettev). 2021 Feb 22;1:5. doi: 10.48130/FR-2021-0005 (PMC11524229; doi:10.48130/FR-2021-0005)
Supplement: Supplementary file 1 — Supplementary data to this article can be found online. [file FR-2021-0005-S1.zip › 10.48130_FR-2021-0005-Suppl-TableS1.docx]

Table S1: RNA sequencing data and corresponding quality control.

| Sample | Raw Reads(M) | Raw Bases(G) | Clean Reads(M) | Clean Bases(G) | Clean Q20(G) | Clean Q30(G) | Clean Length(bp) |
| --- | --- | --- | --- | --- | --- | --- | --- |
| L2025-1 | 41.859 | 6.321 | 38.419(91.8%) | 5.667(89.7%) | 5.571(98.3%) | 5.395(95.2%) | 147.5 |
| L2025-2 | 42.117 | 6.360 | 38.799(92.1%) | 5.724(90.0%) | 5.630(98.4%) | 5.459(95.4%) | 147.5 |
| L2025-3 | 45.758 | 6.909 | 42.295(92.4%) | 6.249(90.4%) | 6.151(98.4%) | 5.972(95.6%) | 147.8 |
| JHY-1 | 37.043 | 5.556 | 34.888(94.2%) | 5.099(91.8%) | 5.003(98.1%) | 4.795(94.0%) | 146.1 |
| JHY-2 | 42.448 | 6.367 | 39.602(93.3%) | 5.754(90.4%) | 5.646(98.1%) | 5.412(94.0%) | 145.3 |
| JHY-3 | 42.377 | 6.357 | 39.740(93.8%) | 5.862(92.2%) | 5.784(98.7%) | 5.634(96.1%) | 147.5 |
