# Supplementary material for: The coloring mechanism of a novel golden variety in Populus deltoides based on the RGB color mode
Source: For Res (Fayettev). 2021 Feb 22;1:5. doi: 10.48130/FR-2021-0005 (PMC11524229; doi:10.48130/FR-2021-0005)
Supplement: Supplementary file 1 — Supplementary data to this article can be found online. [file FR-2021-0005-S1.zip › 10.48130_FR-2021-0005-Suppl-TableS2.docx]

| **Genes** | **Forward Primer** | **Reverse Primer** | |
| --- | --- | --- | --- |
| ***TR10963\|c0_g1(PAL)*** | TCAGTGAACGACAACCCTCTAA | | AGCAGTGAGATTTGATGGCA |
| ***TR23793\|c0_g1(C4H)*** | GCTTATGGTCAAAGAACGCA | | AAGACTGCCCTCCCTATCCT |
| ***TR28152\|c0_g1(CHS)*** | TGCGTTCCTCCGTAAAGAGAT | | CCCACCATTGAATCCAAGTG |
| ***TR13410\|c0_g1(F3H)*** | TGGCGTGAAATAGTGACCT | | AGTATGGCGTTTGAGTCCAGC |
| ***TR30420\|c0_g1(UFGT)*** | CAGTCCTTGCCTTCCCTTT | | GTCGTATGCTTTGATGTTCGTG |
| ***TR19133\|c0_g3(PAO)*** | ATGTTTCTGACCCTTCGC | | GCCATCTTGGAACCACTTA |
| ***TR16078\|c0_g1(CHLH)*** | GCTGCTTCTCATCATTCCA | | GCCCTTGGTGTTGACATT |
| ***TR12009\|c0_g1(HEMD)*** | TCAGCATACACAGGGACCT | | TGCCTCCAGAAAGACAGAG |
| ***TR10569\|c0_g1(HEMC)*** | CTTCCTTGTGCCATTATCG | | AAACCTTAGACGGGTCCTG |
| ***TR33190\|c0_g1(HEME)*** | CCAATCATCCACTCTTCCC | | TCCCTGCTTGCCTCATAA |
| ***TR7353\|c0_g1(ZEP)*** | GATTTAGATGTTGCTGAGGAGG | | GCACCACAGTGACCTTATCC |
| ***TR8948\|c0_g1(PSY)*** | GCACGCTTTATTTGTCCC | | GGGCTAATGAGTGTTCCAGT |
| ***TR15883\|c1_g1(Actin)*** | GGGAAGCGAAGAACTTGATT | | GGTCACTGAAGGAGAAGGGA |

Table S2: List of qRT-PCR's primers
