# Supplementary figures and images for: The coloring mechanism of a novel golden variety in Populus deltoides based on the RGB color mode
Source: For Res (Fayettev). 2021 Feb 22;1:5. doi: 10.48130/FR-2021-0005 (PMC11524229; doi:10.48130/FR-2021-0005)

a

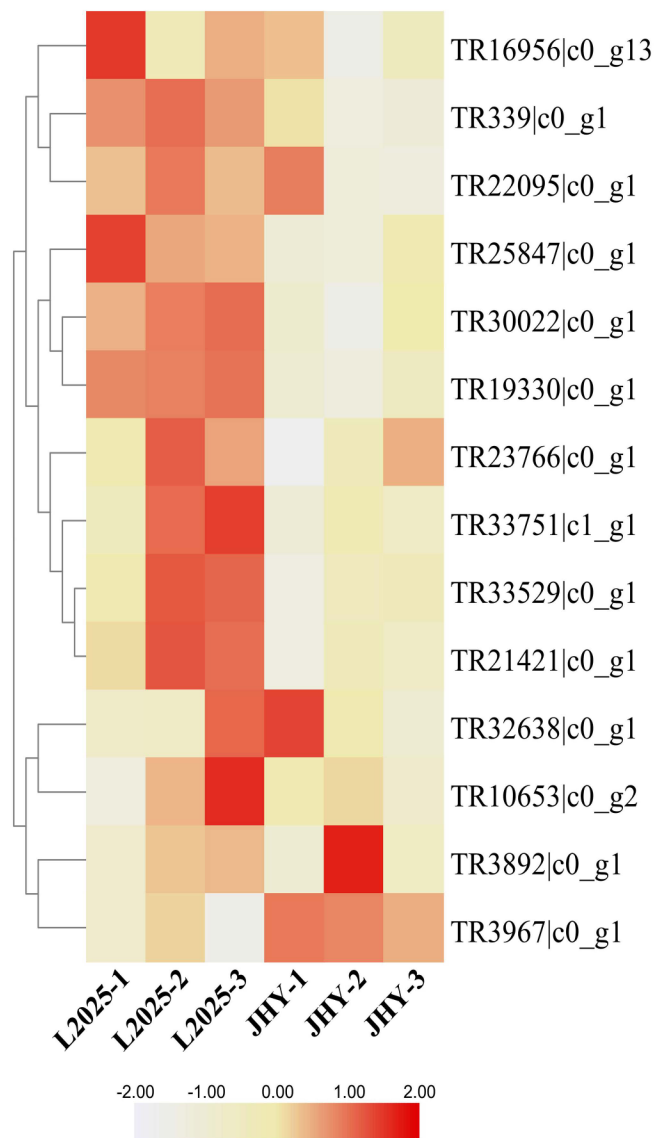

b

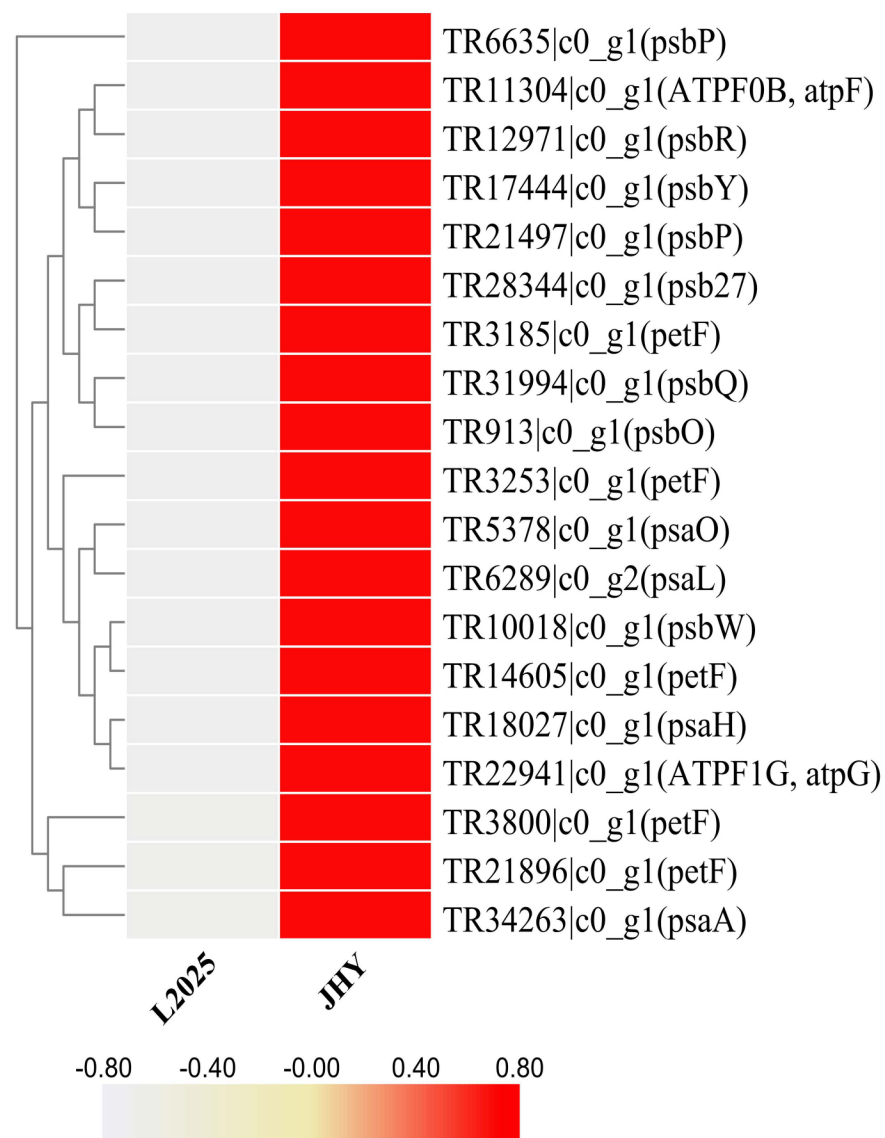

Supplement: Supplementary file 1 — Supplementary data to this article can be found online. [file FR-2021-0005-S1.zip › 10.48130_FR-2021-0005-Suppl-FigureS2.pdf]

**A**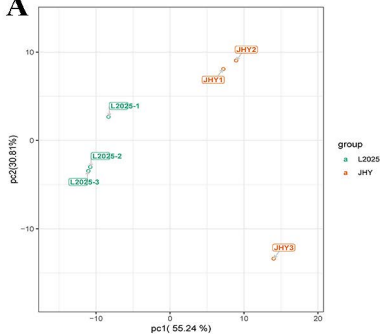**B**

Scores (OPLS-DA)

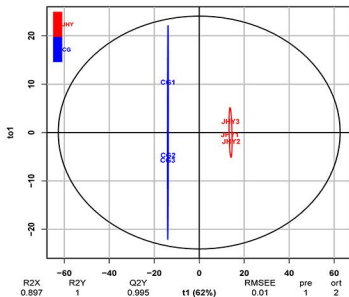**C**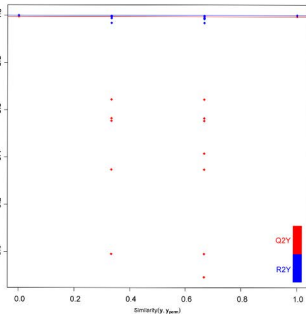

Supplement: Supplementary file 1 — Supplementary data to this article can be found online. [file FR-2021-0005-S1.zip › 10.48130_FR-2021-0005-Suppl-FigureS3.pdf]

**A**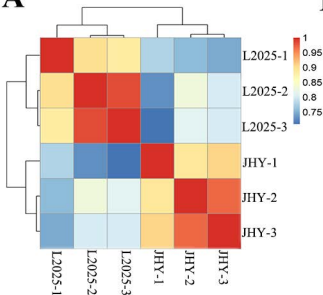**B**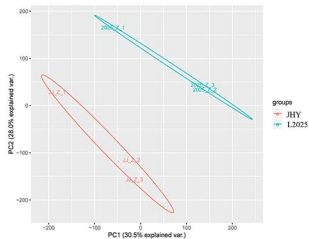**C**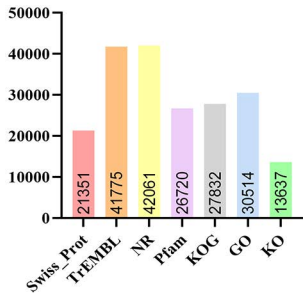**D**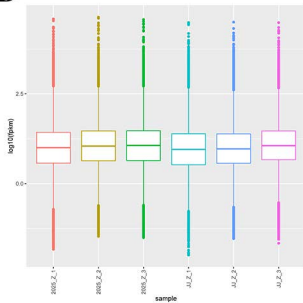

Supplement: Supplementary file 1 — Supplementary data to this article can be found online. [file FR-2021-0005-S1.zip › 10.48130_FR-2021-0005-Suppl-FigureS1.pdf]
